# Beacons and Blockchains in the Mobile Gaming Ecosystem: A Feasibility Analysis

**DOI:** 10.3390/s21030862

**Published:** 2021-01-28

**Authors:** Iakovos Pittaras, Nikos Fotiou, Vasilios A. Siris, George C. Polyzos

**Affiliations:** Mobile Multimedia Laboratory, Department of Informatics, School of Information Sciences and Technology, Athens University of Economics and Business, 104 34 Athens, Greece; pittaras@aueb.gr (I.P.); vsiris@aueb.gr (V.A.S.); polyzos@aueb.gr (G.C.P.)

**Keywords:** distributed ledger technologies (DLTs), interledger, ethereum, hyperledger fabric, internet of things (IoT), location-based mobile gaming, context-aware mobile gaming

## Abstract

We explore the adoption of the Internet of Things (IoT) and Distributed Ledger Technologies (DLTs), such as blockchains, in mobile gaming, focusing on ecosystem expansion and diversification, customer attraction and retention, exploitation of context sensitive and personalized advertisements, and improved monetization of in-game assets. We evaluate the cost and transaction delay of DLTs in a location-based mobile game ecosystem using two types of blockchains (permissioned and permissionless or public), based on defined Key Performance Indicators (KPIs). Our evaluation shows the advantages of using both types of blockchains as well as interledger technologies that combine them. Permissioned blockchains enable high performance, e.g., in terms of throughput and delay, and low cost, while permissionless (public) blockchains, through their transparency, immutability, and openness, support trust and facilitate interactions among unrelated parties. Finally, we show that the combination of IoT devices and DLTs in mobile gaming offers new business opportunities and enables innovative business models for both traditional mobile gaming companies and other participants in the ecosystem, e.g., game players, cafes, malls, and similar establishments, advertising companies, and independent programmers.

## 1. Introduction

Distributed Ledger Technologies (DLTs), and blockchains in particular, constitute a significant baseline platform technology for many applications in various domains, as they have many benefits and intriguing properties. The first use of blockchains was as a financial platform [1]. However, since then many industries, including the energy sector [2,3], supply chains [4,5], and food supply chains [6] in particular, have explored the integration of blockchain technologies in their systems, with a variety of objectives. Another such industry that can potentially significantly benefit from the use of blockchains is the mobile gaming industry [7]. As DLT matures, more and more companies from the gaming industry experiment with integrating blockchains into their games. The first blockchain-based game that became a success was CryptoKitties [8], launched in 2017. Since then, many other and different types of blockchain-based games have been developed, including gambling games [9,10], online casinos [11,12], and trading games [13], among others. Each one of these types of games exploits different attributes and advantages of DLTs.

DLTs provide decentralization, immutability and improve robustness, availability, transparency, and finally trust among participants, enabling untrusting parties to interact and engage with each other, without the need for a trusted third party. When it comes to the mobile gaming industry, blockchains can help in the creation of large gaming ecosystems, involving many and diverse stakeholders that may have conflicting interests, e.g., advertising companies and independent programmers among others.

Nevertheless, these properties come with a cost. The CryptoKitties game revealed some of the inefficiencies of blockchain technology. CryptoKitties attracted a lot of attention (more than 40,000 active users per day), leading to increased traffic on the Ethereum network, resulting in a sixfold increase in pending transactions on the Ethereum blockchain [14]. Furthermore, developers of such games have to face a variety of other issues regarding the performance of their games, such as increased delays, scalability issues, and last but not least, high costs. Therefore, designing a blockchain-based game is not a trivial task and sometimes it can be very challenging, leading to non-scalable and non user-friendly systems.

In this work, we explore different system architectures, combining various types of ledgers, in order to determine a suitable architecture for blockchain-based games, and investigate the relative trade-offs in combining public and private ledger technologies. Key advances in the blockchain technology exploited in this work are: The Ethereum blockchain [15] that introduced Turing-complete smart contracts [16], permissioned ledgers, with the widely-used representative Hyperledger Fabric [17], which allows efficient and high performance operations in consortia, and finally interledger mechanisms [18], which allow hybrid ledger ecosystems, benefiting from the best of both worlds.

In addition, we consider the inclusion of Internet of Things (IoT) devices and technologies in mobile gaming, which can help in the creation of novel and fun mobile games, while enlarging the set of involved parties and creating new business opportunities at the same time.

The IoT is an ecosystem of interconnected devices that can communicate with each other. The increasing number of devices connected to the Internet creates opportunities for new applications, which merge the cyber with the physical world. Gaming companies, and in particular mobile gaming companies, try to take advantage of the many benefits and opportunities that IoT provides in mobile gaming [19]. They try to combine the real with virtual worlds, in order to develop fun augmented or mixed reality games that make players feel that they are personally involved in the game environment, part of which is in the real world. Furthermore, by creating games that combine the physical with the digital world, new business opportunities arise. Cafes, malls, and similar establishments and companies, can participate in such applications, creating or enlarging an ecosystem, by deploying IoT devices in their premises in order to contribute and expand the location-based, context-aware, augmented, or mixed reality gaming experience, as we will discuss below.

In this paper, we present our recent work, undertaken in the context of the H2020 SOFIE project [20], on investigating and evaluating the use of the IoT and DLT in mobile gaming. The contributions of this paper are the following:

- We investigate exploiting the IoT and DLTs in designing an expanding mobile gaming platform that can support an open mobile gaming ecosystem with improved in-game features (e.g., in-game assets), transparent transactions among the various entities (e.g., players, game developers, and advertisers), cross-game and game company assets and value transfers, and player interactions with the real world;
- We define Key Performance Indicators (KPI) for IoT-based, DLT-supported mobile gaming platforms;
- We illustrate the advantages of permissioned and permissionless blockchains in the mobile gaming context and demonstrate the benefits of interledger technology in combining both types of blockchains, creating hybrid environments;
- We evaluate a specific IoT and hybrid DLT mobile gaming platform we designed through a combination of emulation of gaming functions and actual implementations of DLT functionality (e.g., smart contracts) with different combinations of an Ethereum public blockchain [15], a private instance of Ethereum, and a Hyperledger Fabric permissioned blockchain [17].

The remainder of this paper is structured as follows: In Section 2, we present background information about the technologies used in our work. In Section 3, we present the related work in the area, with focus on the gaming industry and how it utilizes blockchains. In Section 4, we describe a particular use case of a mobile game and we introduce its various configurations. In addition, we present the emulation environment, which we developed for its evaluation. The actual evaluation of the use case is presented in Section 5. Section 6 discusses the benefits of the IoT and DLTs in mobile gaming in more general terms. Finally, Section 7 provides our conclusions and directions and plans for future work.

## 2. DLT Background

A blockchain is an append-only ledger of transactions distributed throughout a network of trustless nodes. Hence, they are also referred to as Distributed Ledger Technologies (DLTs). Each block of the blockchain contains a list of validated transactions organized in a Merkle Tree. Transactions are validated by several network nodes and are added in the ledger upon consensus (usually with a Byzantine Fault Tolerant protocol [21]). A blockchain may be public (permissionless), e.g., Ethereum [15], or private (permissioned), e.g., Hyperledger Fabric [17], even though finer distinctions can be made in some cases. Public blockchains can be observed by anyone and anyone can submit transactions and join the network and even participate in the consensus process, while private blockchains can be observed and altered only by the nodes having the right credentials (which implies that some entity specifies the set of possible participants, i.e., gives them permission to join). A tutorial introduction to blockchains and DLTs and their potential applicability in the IoT and intelligent environments in general can be found in the paper by Voulgaris et al. [22]. In this paper we employ two different blockchain technologies: Ethereum and Hyperledger Fabric.

A popular implementation of a public, permissionless blockchain is Ethereum. Ethereum is an open source decentralized computing infrastructure that is capable of executing immutable distributed applications known as smart contracts. Immutability in this context refers to the fact that once a smart contract is deployed, its code cannot be changed. Users interact with a smart contract by sending transactions, which if valid, are appended to the ledger by special nodes known as miners, allowing anybody to retrieve a contract’s execution history and past values of its variables. Users own a public/private key pair that is stored in a wallet and it is used for signing transactions. Transactions are sent to the network by executing a Remote Procedure Call (RPC) on an Ethereum node that may or may not belong to the user.

On the other hand, Hyperledger Fabric is a private, permissioned, blockchain technology, meaning that the membership to the network is controlled. Fabric, like Ethereum, supports the execution of distributed applications, called chaincodes in this case (but for simplicity, in this paper we will refer to chaincodes as smart contracts when we discuss running code on blockchains in general). Chaincode (smart contracts) executed on Fabric can be written in any programming language, including JavaScript and Go, among others. Chaincode is executed simultaneously and in parallel by special nodes on the Fabric network, called endorsing peers. When all the appropriate endorsing peers, specified by the endorsement policy execute a transaction, they send back the transaction result. Eventually, the results are gathered by another special node of the Hyperledger Fabric network, called orderer, which orders the results, creates the block, and broadcasts this block to the Hyperledger Fabric network (i.e., permissioned the blockchain).

## 3. Related Work

Companies in the gaming industry have already integrated blockchains into their products. One of the first blockchain-based games, introduced in 2014, was Huntercoin [23]. Huntercoin is a game where players control a “hunter”, who explores a 2D virtual universe residing within the blockchain. The goal of this game is to collect coins, which can be exchanged with fiat currency. Thus, the blockchain is used mainly for mining, storing, and trading these in-game coins. As we have mentioned earlier, the first blockchain-based game that became a success is CryptoKitties [8]. It is a game where players can buy and “breed” kitties, which are represented by blockchain-based tokens. In particular, the game uses the ERC 721 token standard (http://erc721.org/) to create and manage tokens that represent the in-game assets.

Another game that launched more recently, in February, 2020, is Decentraland [24]. Decentraland is a distributed platform for a shared virtual world that enables players to build on top of it. It uses the Ethereum blockchain and utilizes the ERC 20 token standard (https://eips.ethereum.org/EIPS/eip-20) in order to allow players to trade goods and services provided by themselves. Finally, another recent game that shows the benefits of blockchains in gaming is KotoWars [25]. In this game, players are able to duel each other, using their assets (“kitties”) earned from the CryptoKitties game. This game is of particular interest as it demonstrates how blockchain technology can enable interoperability among different games. It is clear that many games that utilize blockchains have been developed, which take advantage of the features and properties of the DLTs. However, there are not many games or mobile games, that combine DLTs with the IoT. In this paper, we present and evaluate various aspects of a context-aware mobile game that has been developed for the SOFIE project and utilizes both IoT devices and blockchains.

In addition to gaming companies, many research efforts have considered utilizing blockchain technology within gaming. Min et al. [26] survey and categorize existing blockchain-based games according to the properties and features of the blockchain they use, while Min and Cai explore architectures used in blockchain-based games and the security issues that arise [27], and Cai and Wu [28] have proposed a gaming avatar framework that provides interoperability across multiple games and blockchains. On the other hand, Kalra et al. [29] have used blockchain technology, not for the game itself, but for implementing auxiliary functionalities for the games. In particular, they have designed an application that addresses two seemingly different problems in online gaming, cheating and DDoS attacks to game servers.

Although all these efforts illustrate the advantages, as well as the potential for using blockchain in gaming, they do not provide insights about the impact of this technology in gaming applications. In this work, we fill this gap by discussing and evaluating the impact of the inclusion of blockchains to mobile gaming and propose the use of interledger technology in order to exploit both public and private ledgers while achieving the high performance expected in gaming.

This paper summarizes our efforts in evaluating a context-aware mobile game, as part of the H2020 project Secure Open Federation for Internet Everywhere (SOFIE). The goal of the SOFIE project was to federate existing IoT platforms, without requiring any internal change, using distributed ledger and interledger technologies. SOFIE proposed and used four real-life pilots to obtain requirements for the SOFIE architecture and framework components developed and to demonstrate the applicability and effectiveness of the approach. Two of the pilots are described in [30]. One is a food supply chain application capable of certifying the provenance and the conditions of the full path of the food, from field-to-fork through immutable recording of sensors and in general IoT technology (including perhaps exploitation of actuators, e.g., in the form of controlling storage temperature). The second is smart grid applications where electrical power distribution is balanced through targeted electric vehicle charging with the right incentives provided to interest parties through auctions on blockchains.

In this paper, we consider the third real life pilot of SOFIE that utilizes blockchains and IoT technologies in location-based and context-aware mobile gaming. Manzoor et al. [31] describe in detail this location-based mobile game. They present an architecture for such games and then they describe a specific game which they have designed and implemented, investigating the latency and the throughput aspects of the system. The game is a full implementation with a user interface and can be played on real mobile devices, using real Bluetooth beacons or Wi-Fi access points as beacons. Due to their approach, they have to make specific implementation decisions and limit the choices and parameters they investigate, but they also obtain concrete (but technology and solution specific) performance metrics, e.g., for the (mean) time to detect a beacon and the number of players that can be supported.

In our work and in this paper, we use mainly emulation in order to investigate a larger universe and potential applications and mobile gaming ecosystem (but we do actual implementation for all smart contracts and related DLT functionality). This allows us to research more architectural scenarios that jointly utilize public and private blockchain technologies, determine the corresponding trade-offs, and investigate many more performance metrics.

## 4. A Scavenger Hunt Location-Based Mobile Game Ecosystem Emulation

The mobile gaming industry has been considering how to expand the gaming ecosystem. Various ideas have been contemplated and directions proposed. First, improving player trust in the exclusivity, rareness, or even uniqueness of in-game assets provided by gaming companies and traded by players. This is a privileged area for blockchains. Then, if that is secured, the (long-term) value of assets increases and it is felt that players that own such assets should be able to transfer them between games, even across ecosystems of different gaming companies. Again DLTs can play a role here and if the ecosystems use different blockchains, interledger technology provides solutions. Another direction exploited to engage players and programmers and expand games and keep them fresh and adapt them to various communities and interests is the incorporation of the capability of third parties to design ”challenges”. These are specific tasks designed and programmed possibly by independent (to the main gaming company) parties that are attached to the game and expand it. These challenges can bring their own rewards, in particular if there is a general, accessible system of rewards and assets endorsed by the game, such as a blockchain, e.g., Ethereum tokens can play both roles. Finally, advertising is also present in this domain. More interesting is perhaps context-aware ads, perhaps related to the game and the player’s performance, interests, and choices. Enabling advertising companies to conduct campaigns outside the complete control, and perhaps without a-priori knowledge, of the gaming company is an intriguing possibility, potentially supported by open DLTs.

A different direction has been very successfully brought to the forefront by Pokémon Go, one of the most used and profitable mobile apps in 2016. In addition to Augmented Reality, it popularized location-based gaming technology, creating opportunities for brick and mortar stores and other businesses to exploit the digital revolution and mobile gaming in particular to improve their bottom line due to increased foot traffic. On the other hand Pokémon Go also exhibited many other features of mobile gaming we are considering and trying to address through technology in this works: Cheating and hacks by players, the power and presumed arbitrariness of the gaming company, the performance problems of centralization, in particular in the case of targeted denial of service attacks, the low resolution of the location technology, and others. However the success and publicity created by Pokémon Go has intensified interest in location-based and context-aware gaming more generally and in particular in combination with advanced IoT technology availability and affordability.

In order to evaluate how blockchains and IoT technologies perform in demanding mobile gaming applications and their potential impact on expanding gaming ecosystems, we emulated a generalized version of the scavenger hunt location-based mobile game introduced and implemented by Rovio in the EU H2020 project SOFIE.

The game is designed around locations with players incentivized to move from location to location with puzzles presented only when present in the right location and rewards for solving the puzzles. It utilizes IoT beacons or specific Wi-Fi access points or localization, which includes “challenges” and exploits DLTs. A comprehensive description of the mobile game and its architecture is presented in [31]. In a nutshell, the players have to solve riddles using clues to reveal target locations. By solving these riddles, the players are rewarded with points, which they can exchange with rewards and assets. From a high level perspective, the flow of the game is as follows. Initially, the player sees the available nearby riddles (challenges), based on their GPS location. Then, they select one and receive clues that will lead to the first location that the player has to visit. When they arrive at the location of a deployed IoT device, a riddle alongside with some clues are shown on the mobile device. Solving the riddle or performing the appropriate task (e.g., take a picture of a statue located in square X, where X is four blocks away from square Y) will reveal the location of the next IoT device, in order for the player to go there, collect points, and download the next riddle. This procedure continues until the last IoT device is reached.

In addition to the core functionality of the game described above, the specific mobile game provides some additional features. A user is able to skip any challenge, at any time of the game, by viewing an advertisement offered by an advertising company, by paying in in-app tokens, or in fiat currency. Furthermore, a player can get a reward, which can be an asset for the game, by the advertising company if they watch an advertisement. Moreover, the player can at any point of the game redeem points in order to receive rewards given by the game company. The rewards are assets (e.g., a sword or a shield) that can be used in the game or in any other game that uses the same blockchain platform, thanks to blockchain properties, persistence, immutability, and transparency.

The blockchains considered in the design of the game are Ethereum and Hyperledger Fabric for different roles and reasons. Ethereum is a well-known public blockchain (and thus trusted) supporting smart contracts and a widely-accepted cryptocurrency, but has cost and performance limits. Hyperledger Fabric provides high performance, privacy in groups, and a more flexible programming environment, at no monetary and no significant energy cost. A private Ethereum version, without the energy-hungry proof-of-work can be used as an alternative to Hyperledger Fabric. These blockchains are chosen for external and internal gaming functions, respectively. To interconnect these two blockchains, there is a need for an Interledger Gateway (ILG) [18] that handles communication between two ledgers. An ILG is a component that enables interoperability among different DLTs, by transferring information and value between them. In the SOFIE project, an interledger component has been developed (https://github.com/SOFIE-project/Interledger) that enables activity on one ledger, called the *Initiator*, to trigger activity on one or more ledgers, called the *Responder(s)*. This specific interledger component is used in the mobile game in order to allow the communication between the Hyperledger Fabric and public or private Ethereum networks.

For the IoT part of the game, IoT beacons are used as proximity sensors to determine the location of a player, when the user visits the corresponding Point of Interest (PoI). The beacons could be sensed by the user’s smartphone using the Bluetooth Low Energy (BLE) standard, or even Wi-Fi, with different accuracies.

### Emulation Environment and Modeling

In this section, we present the emulation environment that we developed in order to evaluate the performance and other aspects of the scavenger hunt location-based mobile game in more general settings as well as its ecosystem expansion potential. The emulation allows us to easily compare different DLT setups that utilize public (permissioneless) and private (permissioned) blockchains, which would be complicated and difficult with the actual mobile game implementation. Our emulation involves and investigates public ledgers, using a public Ethereum test network, and private ledgers, using a private Ethereum network, or a Hyperledger Fabric instance, allowing us to compare different configurations with the two aforementioned types of blockchains that have different features and trade-offs in terms of transaction cost, latency, transparency, and privacy, among others. Our evaluation through the emulation environment focuses on some system performance metrics (KPIs, defined in the following Section) and does not consider business-oriented metrics such as player satisfaction, gaming experience, and revenue opportunities.

**Figure 1:**
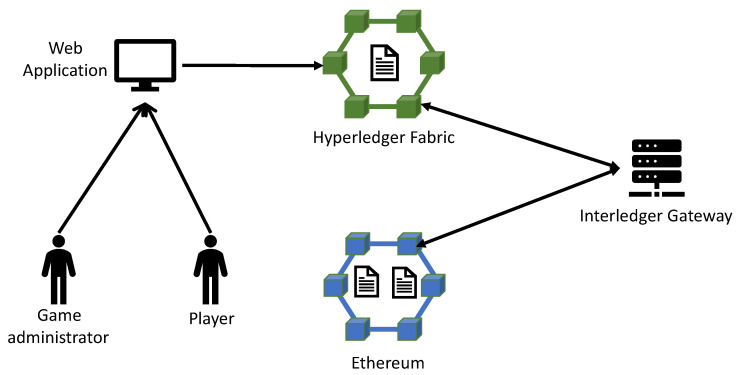
Architecture diagram for the emulation environment involving two blockchains that are interconnected through an Interledger Gateway.

Our developed emulation environment is composed of the following entities and actors (see also Figure 1):

- A Web application that emulates the mobile gaming client;
- The players;
- The game administrator;
- The (public or private) Ethereum blockchain and the corresponding smart contracts;
- The Hyperledger Fabric blockchain and the corresponding smart contracts;
- An Interledger Gateway.

The first component of our system is the mobile gaming client, which is emulated as a Web application implemented in React.js (https://reactjs.org/). Both players and administrators use this Web application to interact with the game, to allow players to play the game, and administrators to modify anything on the game. In order for the actors to interact with the application, they need to own an account in the gaming system, as well as a blockchain wallet account. The latter account corresponds to a public/private key pair for performing transactions on the blockchain. Each challenge/riddle is identified by a unique identifier. In order for a player to select and “play” a challenge, they must choose the appropriate challenge identifier. The solution of the challenge’s riddle is emulated in the application through a “complete” button. The player can skip a challenge by paying some predefined amount in cryptocurrency, or in in-app tokens, or by viewing an advertisement. The in-app tokens are developed using the Ethereum ERC 20 token standard. Finally, advertisements are emulated as functions of a smart contract. Thus, if a player wants to “watch” an advertisement and get the corresponding rewards, they must call the appropriate function of the smart contract from the application.

The main functionality of the scavenger hunt location-based mobile game is emulated by three smart contracts. The first smart contract, named *game smart contract* implements all the functionality related to the challenges and rewards. In particular, it records the challenges on the blockchain. It also records a mapping of players and challenges and whether a particular player has completed the tasks of a challenge or not. Furthermore, the smart contract automatically calculates the points that each player obtains, when they complete the challenge. Moreover, it implements the functions for skipping a challenge. Finally, it implements a function for redeeming the rewards. The second smart contract, called *ads smart contract* contains the functions that emulate the advertisements. It is responsible for checking whether the player “watched” the advertisement or not and provides the corresponding rewards. The third smart contract is the *token smart contract*. The token smart contract mints, burns, and manages the in-app tokens.

**Figure 2:**
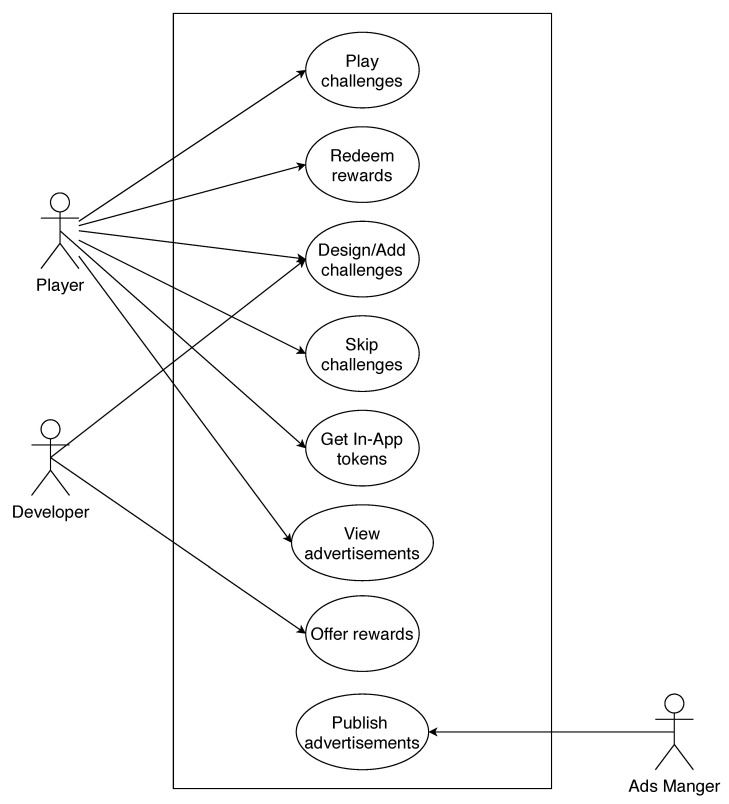
Actors’ interaction with the mobile gaming system.

Figure 1 contains the architecture diagram of our emulation environment. It shows all the components and their interactions, in the case, where the gaming functions are implemented in smart contracts running on different blockchains, which are interconnected through the ILG. In our emulation environment, we have implemented the ILG as one entity that “watches” all the blockchains for emitted events and acts accordingly when it receives one such event. Adding one entity that acts as the ILG is the easiest and typical solution. However, more robust solutions [32] can be considered if needed. The interledger functions are implemented using Node.js and the web3 JavaScript library (https://web3js.readthedocs.io/en/v1.2.9/) in order to interact with the smart contracts. The architecture shown in this figure is one of the four architecture scenarios that we have developed, and in particular is the fourth emulation scenario (see below). Figure 2 presents the actors’ interactions with the emulated gaming system (emulation environment). The diagram shows all the involved actors of the mobile gaming ecosystem, and the functions of the emulated mobile game, they can perform or interact with. The four emulation scenarios that involve different DLTs setup are presented below.

**Scenario 1—One public blockchain, Ethereum:** The first scenario considers a single public Ethereum blockchain. All three smart contracts, which implement the gaming functionality, are deployed on the Rinkeby Ethereum test network (https://www.rinkeby.io).

**Scenario 2—Two types of blockchains, public and private Ethereum:** The second scenario investigates the gains from utilizing two types of blockchains, a public blockchain, and a private/permissioned blockchain. The public blockchain is the Rinkeby Ethereum test network, while the permissioned blockchain is a private instance of Ethereum. The game smart contract is deployed on the private ledger, while the other two smart contracts are deployed on the public ledger. In order for the two ledgers to communicate with each other, there is an ILG, which is responsible for the interconnection of them. ILG “listens” for events on both instances of Ethereum and handles the corresponding functions.

**Scenario 3—One private blockchain, Hyperledger Fabric:** The third scenario considers a single private ledger. In particular, we use Hyperledger Fabric. All smart contracts are deployed on the Fabric blockchain.

**Scenario 4—Two types of blockchains, public Ethereum and Hyperledger Fabric:** Last but not least, the fourth emulation scenario utilizes the two types of blockchains, as in scenario 2, but differs from that scenario in that the private blockchain that is used is Hyperledger Fabric, instead of a private instance of Ethereum.

## 5. Performance Evaluation

To evaluate the performance of the presented mobile game using the emulation environment, we first define the performance metrics that play a crucial role in the performance of the system. The first performance metric we consider is the response time, namely the time required to execute various requests. In this case, where the gaming system utilizes blockchain and IoT devices, the response time is affected by the time that the system performs read and write transactions, and the time that an IoT device needs to detect the players arriving in a particular location. Furthermore, transactions on a public blockchain incur a transaction cost, which in Ethereum is expressed as the cost of gas for executing transactions on the EVM. Thus, since we utilize public Ethereum in mobile game, we should consider the execution cost, measured in gas, as a performance metric. Finally, the last two performance metrics we consider are the throughput and the scalability, since they characterize the volume of transactions, as well as the number of players that the system can support in a given time window.

The aforementioned metrics constitute the Key Performance Indicators (KPIs) and are shown in Table 1. As we have already discussed above, we do not consider business-oriented metrics, and that is why in the table below there are not popular KPIs for mobile gaming, such as Daily Active Users (DAU), Average Revenue Per User (ARPU), and Monthly Active Users (MAU) among others, which are important but addressable only through real implementation. Below, we describe the experiments that we performed, in order to measure all the defined KPIs, and the corresponding results for all the emulation scenarios described in the previous Section.

The smart contracts running on the Ethereum blockchain were written in Solidity, while the smart contracts running on Fabric were written in Node.js. Ethereum smart contracts were tested in the Rinkeby Ethereum test network. On the other hand, for the scenarios that leverage Hyperledger Fabric, we used Fabric v1.4. The network in Hyperledger Fabric consisted of two organizations, having two endorsing peers each. The smart contracts were deployed in all peers. The results presented below are the average value of 10 executions. Furthermore, we calculated the 95% confidence interval for each of the experiments, using the Student’s t-distribution (because the sample size is small, <30).

KPI_1 refers to the public ledger execution cost and is measured in gas units. The third scenario, which utilizes only the Hyperledger Fabric, does not entail an execution cost, since it does not perform any actions on a public ledger. For the same reason, some of the actions in the second and fourth scenario incur zero cost too. Furthermore, some actions in different scenarios have the same implementation and hence also have the same cost. All the actions that involve a transaction on a public ledger and the corresponding execution cost, for all scenarios is shown in Table 2.

From the above table, we observe that for all the actions except of one, the execution cost is smaller in the second and fourth scenarios that involve a public and a private ledger. This happens because these actions involve the interaction of the game smart contract with the ads or the token smart contract. In these scenarios, the game smart contract is deployed on the private ledger, so the actions performed by that smart contract do not incur cost. For this reason, the total amount of cost required for these specific actions is the gas consumed only by the public ledger. We should note here that the execution of a transaction in the private Ethereum consumes private EVM resources. So, we assume that the cost is zero, and that is why we have set the results for these actions as Not Applicable (N/A).

The function with the highest execution cost on the second and fourth scenarios is the function invoked for getting tokens by viewing advertisements. This function involves the invocation of all three smart contracts. The player first calls the function of the game smart contract to alert the system that they want to “watch” an advertisement in order to acquire some tokens, then the game smart contract invokes the ads smart contract, and finally when the ads smart contract finishes the execution, the game smart contract invokes the token smart contract to transfer the tokens to the player’s account. In the first scenario, all these invocations can only be done by one transaction for invoking the game smart contract. Then, the game smart contract will invoke the other two smart contracts internally. In the other two scenarios, we cannot perform this specific action by sending just one transaction, since the smart contracts are deployed on different ledgers, thus we need two more transactions, initiated by the ILG. One transaction to invoke the game smart contract (zero cost), which will trigger an event that will be “caught” by the ILG. Then, the ILG will send a transaction to the ads smart contract. When the execution will finish, it will send a transaction back to the game smart contract to inform it that the user “watched” the advertisement. After that, it can proceed with the payment. The same process is followed with the token smart contract. Thus, for this particular action, the second and the fourth scenario adds an overhead of two more transactions on the public ledger. The above results confirm that using a single public ledger is very costly. Furthermore, the results quantify the gains, in terms of cost that can be obtained by combining a private and a public ledger.

The second KPI refers to response time for write requests. In Ethereum, the transaction delay depends on the block mining time. The average time for mining a new block in Ethereum, i.e., the time required by the Ethereum network to generate and append to the blockchain a new block, is ≈15 s. Therefore, for scenarios that utilize the Ethereum blockchain (public or private) the response time for write transactions is ≈15 s. This time is also affected by other external factors, such as the load of the network, the size of the transaction, and how many transactions can fit in a block. We performed some experiments in the Rinkeby Ethereum test network, to validate that the response time is indeed around 15 s.

On the other hand, in Hyperledger Fabric, in order for a transaction to be added on the ledger, it must get through three phases. These phases are the execution phase, the ordering phase, and the validation phase. From a high level perspective, the transaction flow on Hyperledger Fabric is as follows. Initially, the client sends a transaction proposal. The endorsing peers receive the proposal, execute the transaction, and send back to the client the signed result, as a proposal response (execution phase, no updates made to the ledger). Then, the client broadcasts the transaction proposal and response to the ordering service, which orders the transactions and creates the blocks (ordering phase). Finally, the blocks are broadcasted to all peers, are validated, and each peer appends the new block to the blockchain (validation phase). So, in Hyperledger Fabric, the transaction delay depends on the time that each phase requires. The results, with the confidence intervals, for all scenarios are presented in Table 3.

The next KPI refers to the time needed for the system to respond to non-altering transactions, namely read requests. Read requests in Ethereum do not broadcast or publish anything on the blockchain, and the response is returned instantaneously, since the requests are local. Furthermore, read requests in Hyperledger Fabric follow the same flow as the one described above for the write requests, with the difference that for the read requests the flow ends when the proposal response is returned to the client (i.e., there is not an ordering and validation phase). The results are shown in Table 4.

As we can see from the above table, the response time in scenarios that utilize Ethereum is approximately 1 s, which is not negligible as expected. This occurs because in our emulation environment, we do not own and run an Ethereum (full or light) node (this is the case usually). So, in order to interact with the Ethereum blockchain, we communicate with another node of the network, through Remote Procedure Calls (RPCs). Thus, an additional (network) overhead is introduced. We performed some experiments with local Ethereum, in order to find out the exact value for the response time in non-altering transactions, which is 0.045 s.

The next KPI is the BLE beacon detection time. Functionality related to beacon detection time is not implemented in the smart contracts, hence this time is independent of the ledger transaction time. Furthermore, since this action is independent of the blockchains, our emulation environment does not consider IoT-related actions, thus we do not have produced results about this specific KPI. However, in [31], there is an exhaustive evaluation about the beacon performance for this specific mobile game. Authors calculated that the average delay is 5.8 s.

Next, we have the KPI that is related to throughput, which is defined as the number of transactions per time unit that the system can support. To measure this KPI, we conducted a number of experiments sending write requests to the system, since read requests are local, and they do not record anything on the ledger. Initially, we measured the throughput of private Ethereum. In these experiments, we observed that a block can store up to 74 transactions, related to our game. We also know that a block is mined in 15 s, so the throughput of the system is 4 transactions per second. We should note that the throughput is affected by the number of transactions that can fit in a block. In particular, we measure the transactions that fit in a block by calculating the amount of gas that is packed in the block. So, the throughput might be increased for some other actions that costs less gas. In public Ethereum, a block will contain transactions from the whole Ethereum network, and not only from our game, thus the throughput will be even smaller. On the other hand, in Hyperledger Fabric, we have observed from the configuration file that a block can fit 20 transactions, with a maximum transaction size of 99 MBs. From the experiments we conducted, we measured that a block is added on the ledger in about 100 milliseconds, and that all the transactions related to our use case had a transaction size smaller than 99 MBs. Thus, the throughput of the system is 200 transactions per second. The above results are not affected by the ILG component, since we measure the throughput of each blockchain separately.

**Figure 3:**
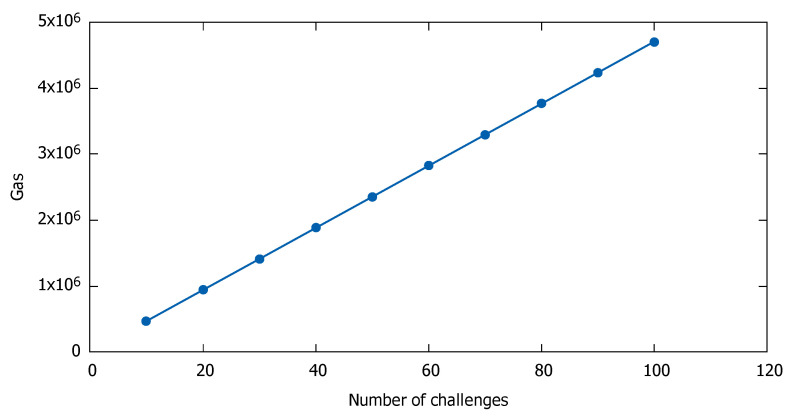
Ethereum gas consumption as a function of the number of challenges.

The last two KPIs are related to scalability, and in particular they show how the execution cost (KPI_6), and the response time (KPI_7) are affected by the number of challenges, or users. Again, as the throughput, scalability is not affected by the ILG or any other component of the system. The cost scalability concerns only scenarios that use the public Ethereum blockchain, which introduces a transaction cost. We defined the cost scalability as the ratio of the cost over the number of challenges. The cost scalability is shown in Figure 3.

From the figure above, we observe that the cost scalability is linear to the number of challenges. Similarly, the cost scalability remains linear, not only to the number of challenges, but also to the number of tokens, points, and any other metric that involves transactions with the Ethereum blockchain.

**Figure 4:**
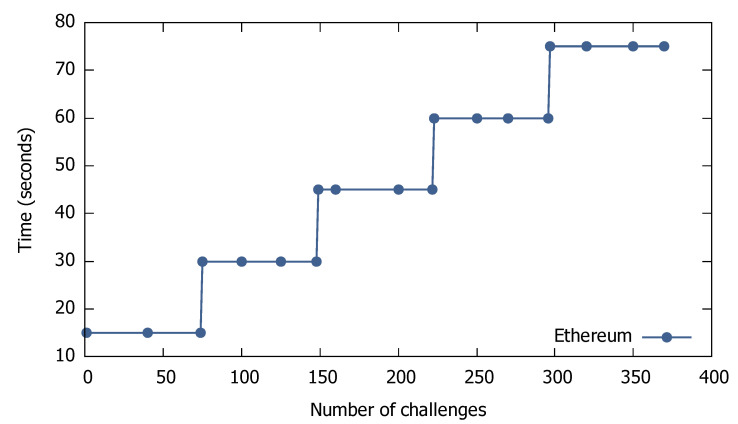
Time scalability for Ethereum-based scenarios.

**Figure 5:**
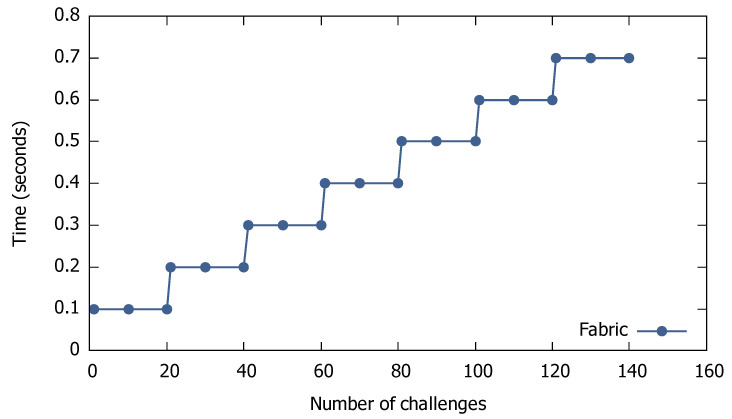
Time scalability for Hyperledger Fabric-based scenarios.

Finally, the last KPI refers to time scalability, which is shown in Figure 4 for the public Ethereum, and in Figure 5 for Hyperledger Fabric, respectively. As for cost scalability, we defined time scalability as the ratio of time over the number of challenges. From the figures, we observe that the time scalability for all types of blockchain is a stepwise function, which macroscopically becomes linear. Again, the time scalability is similar for points, tokens, etc.

All the results for all the KPIs and scenarios are summarized in Table 5. The results in the table are the average of all the corresponding actions of all experiments. Furthermore, for the fourth emulation scenario, which utilizes both public Ethereum and Hyperledger Fabric, the results presented in the table are for both blockchains.

From the above table, it is clear that the use of a public ledger (Scenario 1) is expensive and demonstrates poor performance results, especially in terms of response times and throughput. Using a single private ledger (Scenario 3) is significantly better than using a single public ledger, in terms of cost and performance. However, this alternative lacks some important properties of the public ledger, such as transparency, wide-scale trust, and openness. Thus, the best solution is to combine these two types of blockchains, the private ledger in order to implement all the gaming functionality for which time is a crucial factor, while the public blockchain in order to implement other actions, such as advertisements and asset specific actions, that require higher levels of trust, transparency, and openness in order for the entities to be able to join the ecosystem easily. Therefore, since Hyperledger Fabric demonstrates better performance results than a private instance of Ethereum, the private ledger should be the Hyperledger Fabric, thus the fourth emulation scenario is more suitable than the second, which utilizes public and private Ethereum.

## 6. Discussion

Blockchains have many useful properties, including *decentralization*, *replication*, *transparency*, and *immutability*. These achieve *auditability* and forms of anonymity in some cases. Furthermore blockchains are using based on a (distributed) consensus mechanism: A blockchain can reach consensus without relying on a central, trusted, third party. All these properties and features make DLTs a promising technology for the mobile gaming industry. Blockchains can enhance mobile games with *game and rules transparency*, *assured asset ownership*, *secured asset trade*, *easy and secure asset reusability*, and support for secure and robust *User Generated Content (UGC)*.

Everyone participating in a blockchain network can read all the records stored in the blockchain, as well as the source code of the smart contracts. Thus, if the functionality of a game or the rules of a game are written in a smart contract deployed on the blockchain, everyone, including players, can access and read these data. This is a great improvement compared to legacy systems where usually these data are stored in a private server and are provided to the players by the game company only indirectly, often not even explicitly. So, in a blockchain-based game the players can be sure that the rules of the game will be respected and that the game is played fairly by everyone. One such example is a dice game that generates a random number from one to six, in order to choose a winner. If the functionality that implements the generation of the number is in the smart contract, then the players can audit this process and be sure that no one will cheat, not even the gaming company that provides the game.

Furthermore, different game organizations can join the same blockchain platform and cooperate, in the sense that one company can develop a game based on the content of the game of another company (logic extension and asset reusability), like the KotoWars game [25]. If the assets of a game (e.g., shields and guns) are represented as tokens in a public blockchain, which is open to everyone, then these tokens will “live” in the blockchain network, and not in the server of the specific game. Thus, everyone can also (re)use them outside the game, e.g., in another game that supports the same assets, without the intervention of the first game company.

Blockchain technology can also improve in-game features and solve some of the problems faced by the (mobile) gaming industry. A game that leverages blockchain technology can expand and improve in-app payments using cryptocurrencies. Until recently, mobile games used mostly conventional payment methods that require a third trusted party in order to be reliable. With the use of blockchains and smart contracts, and in particular due to the transparency and openness they provide, in-app payments become transparent without needing a trusted third party. Furthermore, by using smart contracts, which run deterministically (in the EVM) with a source code that cannot be modified (since they are immutable), we can be sure that agreed rules will be respected no matter what.

With regards to advertisements, using the blockchain technology to track in-app advertisements can strengthen this process. Companies from the advertising sector, or individual advertisers, can provide personalized and more importantly context-aware advertisements. In an IoT mobile game (as the one described above), advertising companies can provide advertisements based on the user’s location, in addition to other game context-related information, both macroscopic, e.g., the game or type of game, or the specific state in the game or past history and choices of the player if these become observable to all or specific parties (e.g., the advertisers).

Moreover, as already discussed, a game can leverage a blockchain to provide asset ownership with transparency and consistency of asset rules, and other similar useful properties, leading to trusted and simpler trading transactions of assets among players. More importantly, this can also be done across games. Note that distrust about rare and expensive assets has been a limiting factor in some cases in the past and a key motivating factor for the introduction of DLTs.

Perhaps the most intriguing benefit of blockchains in the (mobile) gaming industry is the support for User Generated Content (UGC). In traditional gaming all the assets of a player in the game belong to the game company, even in games where a player can create their own items. With the help of blockchains, this can change and lead to an open community where any player will be able to create, own, and trade their own items. Furthermore, the items that are generated by the users will be owned by the users themselves, even if they stop playing the game. Additionally, DLTs can secure and strengthen this community through the aforementioned properties, e.g., avoiding incidents of stolen assets. All these properties can help the gaming industry to attract many more players and to engage them more by allowing them to create and trade assets and more generally UGC.

Therefore, it is clear that blockchains have the potential to help in the creation of new and open mobile gaming ecosystems, especially when they are combined with IoT technologies, where many different organizations (e.g., advertising companies, game studios, PoI companies, and others) can cooperate with each other to develop novel, fun, and context-aware mobile games. In our case, it is easy for a cafe, or a mall, or any other company interested in a particular location (PoI), to deploy IoT beacons in its location, design challenges, and add them in the smart contract, without the intervention of a middleman, since there are no barriers to entry, and anyone with an Ethereum address can add a challenge. Of course, the initiator of the game smart contract, typically the gaming company, can introduce conditions to be checked automatically by the smart contract. With a case like that, new business opportunities are created, since the players will be attracted to the PoI in order to complete a challenge and may buy something from a particular store or in general benefit that company. The same also applies for the advertising sector. It is easy to create open ecosystems for the advertisers, in order to add their advertisements in the game, by just adding the URL of the advertisement within the smart contract.

Of course these benefits come with costs. Firstly, and as we show in the previous section, public blockchains involve monetary and communication overhead, which in many cases can be prohibitive. Secondly, the immutability property of the blockchain makes hard for developers to fix bugs in their code, since deployed smart contracts cannot be modified. Thirdly, the openness and transparency of blockchain makes it difficult to protect intellectual property or any other trade secrets.

In this paper we developed an emulation environment in order to evaluate the performance and various other aspects (e.g., open ecosystems) of a location-based mobile game. Through emulation, it is easier and simpler to develop and implement different scenarios, as the ones presented above, which utilize different blockchain technologies. However, our emulation environment has some limitations.

First of all, as we have already mentioned, in our emulation environment, we did not consider IoT-related actions. So, we did not measure the fourth KPI (see Table 1), which is the BLE beacon detection time. However, the detection time of beacons is one of the most important performance metrics for the specific mobile game, since if a beacon takes too long to sense a player’s smartphone and send the appropriate tasks, then the player might be confused and think that they are not in the appropriate location. However, authors in [31] have calculated that the average delay is 5.8 s.

Furthermore, in our evaluation, we did not consider business-related metrics and KPIs, such as DAU and ARPU, since these metrics are addressable only through real implementation. On the other hand, in our emulation we focused on the technologies used in the presented mobile game and how these technologies affect the performance of the system. However, we briefly describe the new business opportunities that arise with a game like the one presented above, (due to the use of IoT and blockchain technology) for PoI companies (restaurants, cafes, and malls among others), advertisers, game developers, and even for players.

Finally, in our emulation environment, we have one application (Web app) that all actors, players, game administrators, and advertisers use to interact with the system. In addition, the entirety of game functionality is implemented in the three smart contracts and the ILG. In real implementation of the game, this is not the case. The game company should have an application that will manage the game (e.g., managing the users accounts), another one to manage the challenges, and so on. However, these (architectural) modifications will not affect the performance results presented above, and the results will remain at the same order of magnitude, as the bottleneck of the system is the blockchain technology.

## 7. Conclusions

In this paper we discussed how DLTs and IoT devices (mostly beacons) could be utilized to enable and expand novel mobile gaming ecosystems and improved in-game features, such as in-app payments without third parties, and personalized and context sensitive advertisements, in an open and secure manner. Furthermore, we described a specific prototype of a location-based mobile game and evaluated it from various aspects based on defined KPIs.

Our evaluation, at a high level, showed the gains that could be achieved in terms of cost and performance, when public and private ledgers are combined. We concluded that using only one permissioned ledger was better in terms of time and cost. However, using two ledgers, a public one and a private one, was better in terms of transparency, trust, and openness. In particular, we claim that using (public) Ethereum for increased trust and convenient payments and Hyperledger Fabric for high performance, scalability, and low cost, is better than using only one type of ledger for many types of games and business logic situations.

Our paper is the first step towards assessing the impact of the IoT and DLTs in the mobile gaming industry. Our findings indicate great potentials and for this reason it is in our future plans to further investigate this field. Our emulations will be complemented by experimentation with real mobile games and more IoT devices, which will provide us with insights about potential deployability issues, as well as, by analytical evaluation through system modeling which can help us evaluate large scale scenarios, as well as predict market directions.

## Figures and Tables

**Table 1:** System key performance indicators for the mobile game.

KPI	Name	Description
KPI_1	Public ledger execution cost	Cost for executing operation on a public ledger
KPI_2	Response time for write requests	Time for the system to respond to write transactions
KPI_3	Response time for read requests	Time for the system to respond to read transactions
KPI_4	BLE beacon detection time	Time that player has to wait between arriving at location and receiving the task
KPI_5	Throughput	Maximum number of transactions per time unit
KPI_6	Cost scalability	Increase of cost as number of challenges increases
KPI_7	Time scalability	Increase of response time as number of challenges increases

**Table 2:** Ethereum virtual machine execution cost (gas).

Actions	Scenario 1	Scenario 2	Scenario 3	Scenario 4
Add challenge	47,050	N/A	N/A	N/A
Begin challenge	52,432	N/A	N/A	N/A
Complete challenge	53,529	N/A	N/A	N/A
Skip challenge by paying	61,867	N/A	N/A	N/A
Skip challenge by in-app tokens	63,877	33,438	N/A	33,438
Skip challenge by viewing ads	53,926	21,462	N/A	21,462
Get tokens by paying	44,199	35,274	N/A	35,274
Get tokens by viewing ads	37,981	56,736	N/A	56,736
Redeem rewards	36,618	35,274	N/A	35,274

**Table 3:** Mean response time units for write requests (confidence intervals).

Actions	Scenario 1	Scenario 2	Scenario 3	Scenario 4
Add challenge	14.55 (±2.48)	14.55 (±2.48)	2.209 (±0.03)	2.209 (±0.03)
Begin challenge	15.38 (±3.14)	15.38 (±3.14)	2.195 (±0.01)	2.195 (±0.01)
Complete challenge	14.14 (±3.57)	14.14 (±3.57)	2.187 (±0.01)	2.187 (±0.01)
Skip challenge by tokens	12.96 (±2.90)	12.96 (±2.90)	2.176 (±0.01)	12.96 (±2.90)
Skip challenge by ads	15.14 (±3.39)	15.14 (±3.39)	2.182 (±0.02)	15.14 (±3.39)
Get tokens	11.12 (±2.05)	11.12 (±2.05)	2.187 (±0.01)	11.12 (±2.05)

**Table 4:** Mean response time units for read requests (confidence intervals).

Actions	Scenario 1	Scenario 2	Scenario 3	Scenario 4
Query points	1.106 (±0.05)	1.106 (±0.05)	0.024 (±0.002)	0.024 (±0.002)
Query challenges	1.085 (±0.04)	1.085 (±0.04)	0.026 (±0.006)	0.026 (±0.006)
Query tokens	1.124 (±0.06)	1.124 (±0.06)	0.021 (±0.001)	1.124 (±0.06)

**Table 5:** System performance evaluation results for all the emulation scenarios.

KPIs	Scenario 1	Scenario 2	Scenario 3	Scenario 4
KPI_1	50,164	36,437	0	0 & 36,437
KPI_2	13.89 s	13.89 s	2.189 s	2.197 & 13.89 s
KPI_3	1.105 s	1.105 s	0.024 s	0.025 & 1.124 s
KPI_4	N/A	N/A	N/A	N/A
KPI_5	4 write tr/s	4 write tr/s	200 write tr/s	Same as scenario 1, 3
KPI_6	Linear	Linear	N/A	Linear
KPI_7	Step-wise	Step-wise	Step-wise	Step-wise

## Data Availability

Not applicable.

## Abbreviations

The following abbreviations are used in this manuscript:

DLT	Distributed Ledger Technology
IoT	Internet of Things
PoI	Point of Interest
URL	Uniform Resource Locator
KPI	Key Performance Indicator
BLE	Bluetooth Low Energy
RPC	Remote Procedure Call
ILG	Interledger Gateway
SOFIE	Secure Open Federation for Internet Everywhere
EVM	Ethereum Virtual Machine
MB	Megabyte
N/A	Not Applicable
UGC	User Generated Content
